# Mechanisms by which SNX-BAR subfamily controls the fate of SNXs’ cargo

**DOI:** 10.3389/fphys.2025.1559313

**Published:** 2025-03-12

**Authors:** Yaolin Long, Yang Li, Jin Xue, Wanqing Geng, Mingxia Ma, Xiaohui Wang, Li Wang

**Affiliations:** ^1^ Basic Medical Research Center, Shanxi Medical University, Taiyuan, Shanxi, China; ^2^ Department of Ophthalmology, Shanxi Medical University Second Affiliated Hospital, Taiyuan, Shanxi, China

**Keywords:** SNX-BAR subfamily, signal transmission, cargo sorting, endosome, autophagy, ubiquitin

## Abstract

The SNX-BAR subfamily is a component of the sorting nexins (SNXs) superfamily. Distinct from other SNXs, which feature a PX domain for phosphoinositide binding, the SNX-BAR subfamily includes a BAR domain that induces membrane curvature. Members of the SNX-BAR subfamily work together to recognize and select specific cargo, regulate receptor signaling, and manage cargo sorting both with and without the involvement of sorting complexes. They play a crucial role in maintaining cellular homeostasis by directing intracellular cargo to appropriate locations through endo-lysosomal, autophagolysosomal, and ubiquitin-proteasome pathways. This subfamily thus links various protein homeostasis pathways. This review examines the established and hypothesized functions of the SNX-BAR subfamily, its role in intracellular protein sorting and stability, and explores the potential involvement of subfamily dysfunction in the pathophysiology of cardiovascular and neurodegenerative diseases.

## 1 Introduction

Cells rely on a multitude of transmembrane proteins, along with their associated proteins and lipids (such as signaling receptors, ion channels, and polar markers), collectively referred to as “cargo,” to interact with their environment. Endosomes, crucial metabolic centers in eukaryotic cells, dictate the fate of endocytic cargo and play a key role in maintaining cellular homeostasis during material exchange (Antonescu et al., 2014; Gilleron et al., 2019) and information transfer (Naslavsky and Caplan, 2018). Once internalized, cargo is sorted through the endocytic network and typically follows one of two paths: it may be recycled to various organelles (Cullen and Steinberg, 2018a) or, in some cases, transported to the trans-Golgi network (TGN) or recycled endosomes and returned to the plasma membrane (PM) via the secretory pathway (Doherty and McMahon, 2009; Repnik et al., 2013). Alternatively, cargo labeled with ubiquitin is encapsulated in intraluminal vesicles (ILVs), which are then budded off from sorting endosomes and ultimately delivered to lysosomes for degradation (Schoneberg et al., 2017a; McCullough et al., 2013; Schoneberg et al., 2017b; Simonetti et al., 2023).

**FIGURE 1:**
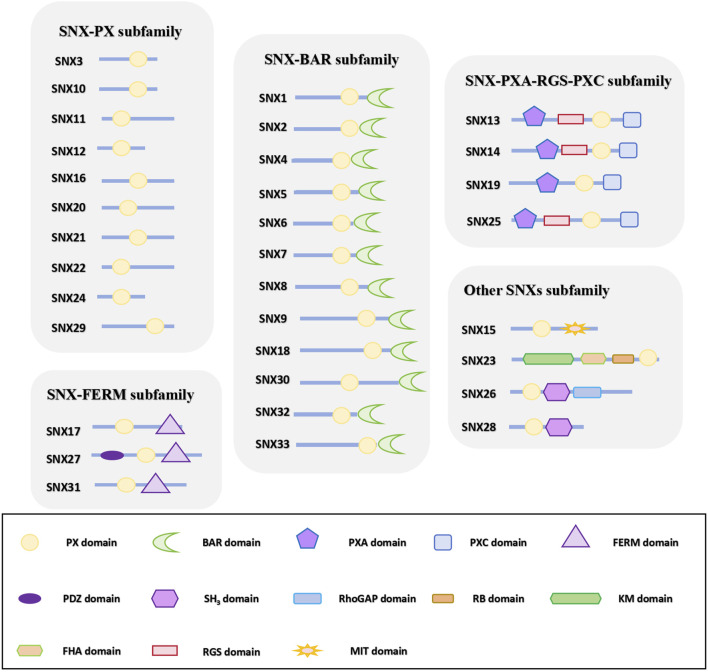
SNXs subfamily classification according to domain organization. The 33 identified members of the SNX family are classified into five subfamilies based on their domain architecture. Some members, featuring distinct domains that do not align with the other subfamilies, are assigned to a separate category. All SNX family members harbor a conserved PX domain, with additional domain variations observed across different subfamilies. PX domain: phagocyte oxidase (phox) homology domain; BAR domain: BinAmphiphysin/Rvs domain; PXA domain: PX-associated domain A; PXC domain: PX-associated domain C; FERM domain: protein 4.1/ezrin/radixin/moesin domain; PDZ domain: postsynaptic density 95/discs large/zonula occludens domain; SH_3_ domain: Src Homology 3 domain; KM domain: kinesin motor domain; RB domain: Rab5-binding domain; FHA domain: forkhead associated domain; MIT domain, microtubule interacting and trafficking domain; RGS: regulator of G-protein signaling domain.

The SNX family, a highly conserved and diverse group of membrane-associated proteins, is vital for regulating the equilibrium of cargo circulation, retrograde transport, and degradation (Hanley and Cooper, 2020). Currently, 33 mammalian SNXs have been identified (Cullen and Korswagen, 2011) and classified into five subfamilies based on their domains: SNX-PX, SNX-BAR (Bin/Amphiphysin/Rvs), SNX-FERM (protein 4.1/ezrin/radixin/moesin), SNX-PXA-RGS-PXC, and other unique SNX subfamilies (Gallon and Cullen, 2015a; Zhang et al., 2018) (Figure 1). Among these, SNX-BARs are the most prevalent and crucial for recycling from endosomes to the TGN and plasma membrane (van Weering et al., 2012a; Wassmer et al., 2007). Studies have demonstrated that the involvement of SNX-BARs in endosomal recycling is dependent on the mammalian retromer complex [VPS26A (or VPS26B)/VPS35/VPS29], which is responsible for cargo recognition. SNX-BARs facilitate membrane remodeling and the formation of tubules and vesicles for cargo transport (Steinberg et al., 2013), thus assisting in the recirculation of endosomes to the PM. While often associated with Retromer-related SNX-BARs (Cullen and Steinberg, 2018b; Yong et al., 2020a), some studies indicate that SNX-BARs can also function independently of retromer in membrane remodeling and cargo sorting (Simonetti et al., 2017a; Kvainickas et al., 2017a; Seaman, 2021; Wang et al., 2018a; McNally and Cullen, 2018).

Maintaining cellular homeostasis involves protein quality control mechanisms that eliminate misfolded, damaged, or redundant proteins and organelles through three primary pathways: the endosome-lysosome pathway, the autophagy-lysosome pathway (ALP), and the ubiquitin-proteasome pathway (UPS) (Chen et al., 2011; Wang and Le, 2019; Pohl and Dikic, 2019). This review will discuss how distinct members of the SNX-BAR subfamily regulate these three protein quality control pathways to maintain cellular homeostasis.

## 2 SNX-BAR subfamily domain and biochemical characteristics

Cell signaling relies on the aggregation of proteins within specific modular regions, which imparts distinct activities or functions to the cell (Mayer, 2015). The SNX-BAR subfamily, as illustrated in Figure 1, includes SNX1, SNX2, SNX4, SNX5, SNX6, SNX7, SNX8, SNX9, SNX18, SNX30, SNX32, and SNX33 (Yong et al., 2020b). In addition to the common PX and BAR domains, SNX9, SNX18, and SNX33 also possess SH3 domains.

### 2.1 PX domain (family characteristics)

The SNX family consists of peripheral membrane proteins involved in protein sorting and transport, all of which feature a shared PX (Phox) domain (Gallon and Cullen, 2015b; Cullen, 2008a). The PX domain was initially identified in the NADPH oxidase subunits p40Phox and p47Phox and has since been observed in various other proteins, including SNX1 (Ponting, 1996). This domain is characterized by three α-helical chains and three anti-parallel β-strands, comprising approximately 100–130 residues (Bravo et al., 2001). A conserved sequence within the PX domain forms a positively charged, proline-rich ring that binds to the negatively charged phosphate groups of phosphoinositides (PIPs) (Seet and Hong, 2006). This interaction provides recruitment signals and facilitates allosteric regulation of different peripheral membrane proteins. The PX domain can bind various phosphoinositides (Teasdale and Collins, 2012), with phosphatidylinositol 3-phosphate (PtdIns3P) being a primary lipid target for PX-domain proteins in mammals (Lucas et al., 2016a). Additionally, the PX domain serves as a protein interaction module. For example, the crystal structures of SNX3 in complex with the VPS26 and VPS35 subunits of the retromer complex demonstrate that the PX domain directly mediates these protein interactions (Lucas et al., 2016b). Furthermore, the PX domains of SNX-BAR subfamily members SNX5 and SNX6 have been shown to interact directly with proteins from the bacterial pathogen *Chlamydia trachomatis* (Aeberhard et al., 2015; Mirrashidi et al., 2015).

### 2.2 BAR domain (special)

The SNX-BAR subfamily features a unique Bin/amphiphysin/Rvs (BAR) domain at the carboxyl terminus. This BAR domain, located adjacent to the PX domain (Carlton et al., 2004a), enables the SNX-BAR subfamily to recognize and interact with various membrane properties—such as curvature, lipid composition, and cargo density—allowing them to traverse between cytoplasmic and endocytic network membranes (Carlton et al., 2004b; van Weering et al., 2012b; Pylypenko et al., 2007). The BAR domain plays a crucial role in membrane morphodynamics, acting as a membrane-binding domain that senses membrane curvature and promotes membrane tabulation (Takei et al., 1999). It binds to membrane surfaces with specific curvatures (Peter et al., 2004) and facilitates dimerization of SNX-BAR proteins through internal interaction sites (Dislich et al., 2011). Hydrophobic and charged interactions among dimers restrict the formation of functional SNX-BAR homodimers or heterodimers (Sierecki et al., 2014). For instance, homodimers of SNX9, SNX18, and SNX33 are associated with the plasma membrane, while SNX8 homodimers localize to endosomes (van Weering et al., 2012c). Additionally, ESCPE-1, composed of SNX1/SNX2 and SNX5/SNX6/SNX32 heterodimers, is involved in endosome-to-Golgi (TGN) recovery and endosome-to-plasma membrane recycling (van Weering et al., 2012d). Conversely, heterodimers of SNX4:SNX7 and SNX4:SNX30 are implicated in autophagy biogenesis (Anton et al., 2020a).

### 2.3 SH_3_ domain (non-major domain)

The BAR domain is rarely found in isolation; it is most commonly associated with the SH_3_ (Src Homology 3) domain, aside from the PX domain (Carman and Dominguez, 2018). The SH_3_ domain, comprising 60 amino acids, is a prominent protein interaction region found in signaling proteins (Dionne et al., 2022). It regulates and participates in various cellular processes, including intercellular signaling, protein transport, and degradation (Kaneko et al., 2008; Tatarova et al., 2012). The SH_3_ domain often contributes to protein complex formation through interactions with other protein regions or through isomeric regulation, thereby stabilizing interactions mediated by other domains of the host protein (Dionne et al., 2021).

## 3 SNX-BAR subfamily and endosome-lysosome pathway

The endosome-lysosome pathway refers to the process by which cells degrade waste materials and damaged proteins through the fusion of endosomes with lysosomes. Research indicates that in mammalian cells, approximately 50%–180% of the plasma membrane surface area cycles through endocytosis and exocytosis every hour (Steinman et al., 1983). Cellular contents and membranes are delivered to early endosomes (EEs), also known as sorting endosomes (SEs), in the peripheral cytoplasm via primary endocytic vesicles. EEs accumulate cargo and either recycle directly to the plasma membrane or through circulating endosomes in the perinuclear region. During this process, EEs mature into late endosomes (LEs), which then fuse with acid hydrolase-rich transport vesicles from the Golgi complex to form a transient organelle, the endolysosome. Under the influence of a proton pump, lysosomes mature and actively degrade their contents for cellular reutilization. This process is essential for protein quality control and maintaining cellular homeostasis (Huotari and Helenius, 2011).

**FIGURE 2:**
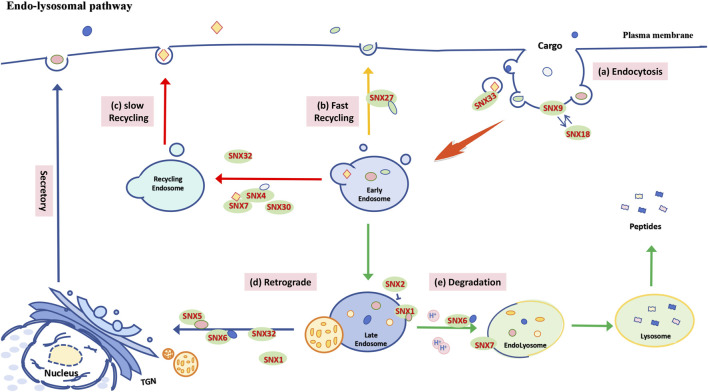
Functional schematic of the SNX-BAR subfamily in the endosome-lysosome pathway. **(A)** Plasma membrane endocytosis: SNX9 and SNX18 regulate cargo internalization through clathrin, while SNX33 is involved in the endocytosis of APP. **(B)** Fast recycling pathway: After cargo is internalized into cells via primary endocytic vesicles, it is delivered to early endosomes (EE) and then recycled back to the plasma membrane through a recycling endosome pathway, which may be mediated by different SNXs in either direct or perinuclear regions. In mammalian cells, the SNX27-mediated rapid endosomal recycling pathway promotes the swift return of cargo to the cell surface. **(C)** Slow recycling pathway: SNX4, through heterodimerization with SNX7 and SNX30, facilitates the transport and recycling of ATG9A and TfR from the plasma membrane to the endosome. **(D)** Retrograde transport: Following cargo accumulation, EE converts into late endosomes (LE), and some internalized cargo is transported to the Golgi apparatus or plasma membrane via retromer complexes or recycling complexes, with assistance from specific SNXs (e.g., SNX1, SNX5, SNX6, SNX32). **(E)** Endolysosomal degradation: A portion of the cargo is sorted by SNXs (such as SNX1, SNX6, SNX7) and fuses with transport vesicles originating from the Golgi complex, which are rich in acidic hydrolases, to form endolysosomes for degradation. Notably, SNX1 and SNX2 exhibit antagonistic roles in regulating the sorting of PAR1 to lysosomes.

The BAR domain of the SNX-BAR subfamily plays a crucial role in forming and stabilizing the tubular subdomains of endosome-mediated cargo recovery (Cullen, 2008b). Additionally, it is involved in regulating protein degradation, endosome-Golgi repair, and endosomal system recycling (Wang et al., 2018b). Besides targeting ubiquitin-labeled goods for lysosomal degradation via the endosomal sorting complex required for transport (ESCRT) (Cullen and Steinberg, 2018c), other internalized materials can be directed to the Golgi apparatus or plasma membrane through reverse transport or recycling complexes, assisted by SNXs and the actin-remodeling WASH complex (Chen K. E. et al., 2019). Within the SNX-BAR subfamily, SNX5 facilitates the retrograde transport of monoamine transporters (VMAT) from endosomes to the Golgi reticulum, where they are assembled into dense core vesicles (Xu et al., 2022) via adapter protein 3 (AP-3) (Figure 2D). SNX9 and SNX18 are involved in plasma membrane endocytosis pathways, including clathrin-mediated protein aggregation, and can functionally compensate for each other (Figure 2A) (Park et al., 2010). SNX32 binds to the immunoglobulin superfamily member Basigin (BSG) via its PX domain, promoting its transport to the cell surface to maintain glial homeostasis (Figure 2D) (Sugatha et al., 2023). Abnormal processing of amyloid precursor protein (APP) results in amyloid beta peptide (Aβ), a pathological marker in Alzheimer’s disease (Zhang et al., 2019). BACE1, involved in APP hydrolysis, is regulated by SNX6, which modulates its retrograde transport (Figure 2D) (Okada et al., 2010), while SNX33 affects APP endocytosis (Takada-Takatori et al., 2019) and α-secretase cleavage of APP (Figure 2A) (Schobel et al., 2008). Furthermore, studies have shown that the binding of SNX4 with SNX7 or SNX30 complexes, or with the retromer complex, facilitates the transport and recycling of autophagy-related protein 9A (ATG9A) and transferrin receptor (TfR) from the plasma membrane to endosomes. (Figure 2C) (Anton et al., 2020a) (Cullen, 2008c). Recent studies also highlight an independent role of SNX-BAR proteins in autophagy, detailed in Section 3.

In mammalian cells, cargo can be recycled to the cell surface via SNX4 (Figure 2C) and SNX27-mediated (Figure 2B) endocytic pathways, while other SNX-BAR family members assist in sorting cargo to lysosomes for degradation (Mallet and Maxfield, 1999; Goldenring, 2015). For example, SNX1 mediates the sorting of protease-activated receptor 1 (PAR1) to lysosomes independently of the reverse transport complex, whereas SNX2 may indirectly regulate PAR1 sorting by affecting SNX1 localization (Figure 2D) (Gullapalli et al., 2006). SNX1 also binds to the epidermal growth factor receptor (EGFR), enhancing its lysosomal degradation (Figure 2E) (Kurten et al., 1996). Furthermore, SNX6-mediated endolysosomal degradation of the tumor suppressor p27 (Kip1) contributes to cell cycle progression (Figure 2E) (Fuster et al., 2010a). SNX4 inhibits BACE1 transport to lysosomes, leading to Aβ accumulation (Figure 2C) (Muller et al., 2017), while overexpression of SNX7 improves APP lysosomal degradation and reduces Aβ production (Figure 2E) (Xu et al., 2018).

In summary, members of the SNX-BAR subfamily play crucial roles in the endocytosis of various cargo, both fast and slow recycling, retrograde transport, and endolysosomal degradation, thereby contributing significantly to cellular homeostasis and protein quality control.

## 4 SNX-BAR subfamily and the autophagy-lysosomal pathway (ALP)

The autophagy-lysosome pathway refers to the process by which cells transport long-lived, highly conserved proteins, dysfunctional or redundant organelles, and protein aggregates into the bilayer membrane of the autophagosome, which are then delivered to the lysosome for degradation. This process efficiently supplies energy and raw materials, thereby sustaining cellular homeostasis (Klionsky, 2007; Lamb et al., 2013). Autophagy is triggered in eukaryotic cells by various external factors (e.g., nutrient deprivation, hypoxia, ischemia) and internal factors (e.g., organelle aging, protein misfolding, DNA damage) that disrupt intracellular homeostasis. It is a critical adaptive mechanism that helps cells manage stress and maintain homeostasis (Dikic, 2017a; Galluzzi et al., 2017). Notably, SNX-BAR proteins operate independently of the reverse transport complex during autophagy (Simonetti et al., 2017b; Kvainickas et al., 2017b). In response to physiological stress, SNXs reposition to participate in macroautophagy. Cargo is encapsulated by autophagosomes through selective or non-selective mechanisms, with membrane extension occurring via contributions from various sources, including the plasma membrane and Golgi apparatus (Guo et al., 2012).

**FIGURE 3:**
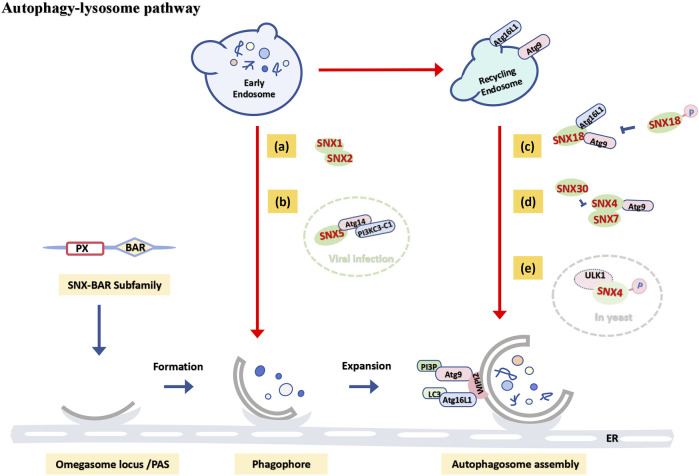
Functional schematic of the SNX-BAR subfamily in the autophagy-lysosome pathway. When cargo is positioned in early endosomes or delivered to recycling endosomes: **(A)** SNX1 and SNX2 cooperate to regulate endosomal tubule formation, participating in starvation-induced autophagy. **(B)** In virus-induced autophagy, SNX5 interacts with the PI3KC3-C1 complex to initiate autophagosome formation (green dashed circle). **(C)** SNX18 positively regulates autophagy by promoting the transport of ATG9A and ATG16L1 to autophagosome assembly sites, though phosphorylation at the S233 site negatively regulates its autophagic function. **(D)** SNX4-SNX7 and SNX4-SNX30 heterodimers jointly regulate autophagosome assembly by controlling ATG9 transport. **(E)** In yeast models, SNX4 may be phosphorylated by ULK1, diverting it from its endosomal sorting function and inducing autophagy (gray dashed circle).

The PX domain of SNX family members binds to different phosphoinositides (PIPs), including PI(3)P, which associates with ATG8 (LC3) to initiate autophagosome membrane formation (Dooley et al., 2014). Members of the SNX-BAR subfamily possess a BAR domain that induces membrane curvature, facilitating the recruitment of autophagy-related proteins and aiding in the extension and closure of the autophagosome membrane (Suarez et al., 2014; Blood and Voth, 2006). Extensive research indicates that PI(3)P-binding BAR domain proteins are closely linked to autophagy biogenesis (Feng et al., 2020; Rodgers et al., 2022). In yeast, the autophagosome assembly site is known as the phage assembly site (PAS) (Hollenstein and Kraft, 2020). Loss of SNX4 impairs PAS formation and delays the autophagy response (Popelka et al., 2017). Recent phosphoproteomic studies in yeast have identified SNX4 as a direct substrate of Atg1/ULK1 (Hu et al., 2019), suggesting that SNX4 phosphorylation may shift its role from endosomal sorting to autophagy induction (Figure 3E). SNX18, a positive autophagy regulator, facilitates the transport of ATG9A and ATG16L1 from cycling endosomes to the autophagosome assembly site by recruiting Dynamin-2 to promote membrane elongation (Soreng et al., 2018). However, phosphorylation of SNX18 at S233 negatively regulates its autophagy function (Figure 3C) (Knaevelsrud et al., 2013).

Autophagy induced by viral infection represents a specialized type of autophagy. Genome-wide RNA interference screenings have revealed that SNX5 is essential exclusively for virus-induced autophagy. Following viral infection, SNX5 interacts with Beclin1 and the Class III phosphatidylinositol 3-kinase (PI3KC3) complex 1 (PI3KC3-C1), which includes ATG14, at early endosomes containing virions. This interaction increases PI3KC3-C1 kinase activity and recruits PI3P and WIPI2 to these endosomes, Initiates the first stage of autophagosome formation, which is the formation of the isolation membrane. Deletion of SNX5 increases cell susceptibility and mortality to viral infection *in vitro* (Figure 3B) (Dong et al., 2021), indicating that enhancing SNX5 expression and autophagy induction may be crucial for the immune response to viral infections.

Members of the SNX-BAR subfamily can function both independently and in heterodimer complexes. For instance, Studies have shown that SNX1 and SNX2 cooperatively induce and regulate the involvement of endosomal tubules in the formation of the isolation membrane (Figure 3A) (Da et al., 2023), with a strong association with starvation-induced autophagy (Da and Morel, 2023). SNX4 is necessary for effective lipidation of LC3 and autophagosome assembly in mammalian cells, while SNX4-SNX7 heterodimers regulate autophagosome assembly by controlling ATG9 transport (Figure 3D) (Anton et al., 2020b). Future research may explore how variations in the balance between heterodimers like SNX4-SNX7 and SNX4-SNX30 affect early autophagy stages.

In summary, the SNX-BAR subfamily can maintain cellular homeostasis primarily through involvement in the biogenesis of autophagosomes, influencing the autophagy-lysosome pathway, independent of retromer transport complexes.

## 5 SNX-BAR subfamily and ubiquitin-proteasome pathway (UPS)

The ubiquitin-proteasome pathway is the primary proteolytic route for misfolded, damaged, and short-lived proteins. Ubiquitin-tagged proteins are typically targeted for degradation by the proteasome or lysosome, thereby regulating protein quality control and maintaining cellular homeostasis (Budenholzer et al., 2017; Bochtler et al., 1999). Ubiquitin (Ub) is a 76-amino acid protein with a molecular weight of approximately 8.5 kDa, ubiquitously present in eukaryotic cells. It forms a covalent bond between the carboxyl group (-COOH) of its C-terminal glycine and the amino group (-NH2) of substrate lysine (Sun and Chen, 2004). Ubiquitination is a common post-translational modification where E1 ubiquitin-activating enzyme, using ATP energy, forms a UB-E1 complex with ubiquitin. This complex then transfers ubiquitin to E2 ubiquitin-conjugating enzyme, forming the UB-E2 complex through transesterification. E3 ubiquitin ligase subsequently attaches the UB-E2 complex to specific target proteins. The type of linkage and the length of the ubiquitin chains confer various biological functions (Akutsu et al., 2016; Dikic, 2017b).

**FIGURE 4:**
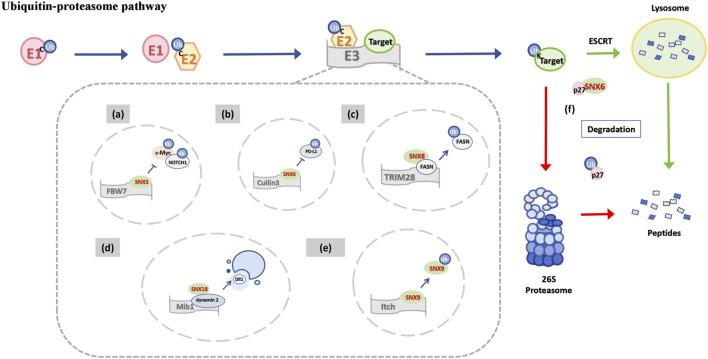
Functional schematic of the SNX-BAR subfamily in the ubiquitin-proteasome pathway. E3 ligases recognize specific substrates and catalyze the covalent attachment of ubiquitin molecules to mark substrates for proteasomal degradation. In this process: **(A)** SNX5 interacts with FBW7 (E3), reducing the ubiquitination and subsequent proteasomal degradation of downstream oncogenic proteins mediated by FBW7. **(B)** SNX6 interacts with Cullin3 (E3) to downregulate Cullin3-mediated ubiquitination and degradation of PD-L1. **(C)** SNX8 directly binds FASN and recruits TRIM28 (E3), thereby promoting the ubiquitination and degradation of FASN. **(D)** Mib1 (E3) regulates SNX18 recruitment of dynamin 2 in a ubiquitin ligase activity-dependent manner, promoting the endocytosis of Dll1. **(E)** Itch (E3) interacts with SNX9 through its PRD domain, mediating the ubiquitination and degradation of SNX9. **(F)** p27 is dual-regulated by both the ubiquitin-proteasome pathway (red arrow) and the SNX6-mediated endolysosomal pathway (green arrow).

Numerous studies have shown that sorting nexins (SNXs) influence proteasome activity and substrate degradation through several mechanisms. This discussion focuses on the mechanisms involving the SNX-BAR subfamily. These mechanisms include inhibiting the ubiquitination of protein substrates and modulating ubiquitin-specific factors. For example, FBW7, an E3 ubiquitin ligase, interacts with SNX5 to reduce the ubiquitination and degradation of cancer-associated proteins such as c-Myc, NOTCH1, and Cyclin E1, thereby promoting the progression of head and neck squamous cell carcinoma (HNSCC) (Figure 4A) (Cai et al., 2019). Similarly, SNX6 downregulates Cullin3-mediated ubiquitination and subsequent degradation of programmed death ligand 1 (PD-L1) by binding to Cullin3, thus enhancing cancer cells’ ability to evade immune surveillance (Figure 4B) (Ghosh et al., 2021). SNX8 interacts directly with fatty acid synthase (FASN) to promote its ubiquitination and proteasomal degradation by recruiting TRIM28, an E3 ubiquitin ligase, and enhancing TRIM28-FASN interactions (Figure 4C) (Hu et al., 2021). Additionally, Mib1, an E3 ubiquitin ligase, regulates SNX18 recruitment of guanosine triphosphate (GTP) - dynamin 2 in a ubiquitin ligase-dependent manner, facilitating the endocytosis of Delta-like 1 (Dll1) and enabling effective Notch signaling for normal development and tissue homeostasis (Figure 4D) (Okano et al., 2016).

In addition to their roles in UPS regulation, SNXs themselves are regulated by UPS mechanisms. Deubiquitinating enzymes (DUBs) modulate substrate activity and abundance by removing ubiquitin-bound proteins (Sowa et al., 2009). For instance, DUBs have been reported to increase stability by interacting with SNX3 (Boulkroun et al., 2008a) and SNX27 (Stangl et al., 2019). The E3 ubiquitin ligase Itch, a member of the NEDD4 family, regulates intracellular levels of SNX9 through interaction with a proline-rich domain (PRD), mediating SNX9 ubiquitination and degradation (Figure 4E) (Baumann et al., 2010). Although reports on the regulation of SNX-BAR subfamily members by UPS and the activities of E1 and E2 enzymes are limited, they support the hypothesis of mutual interaction between SNX-BAR subfamily members and the UPS.

Notably, selective proteolysis is primarily mediated by both the UPS and the autophagy-lysosomal pathway (ALP), and recent research has highlighted their functional interrelation (Korolchuk et al., 2010; Ji and Kwon, 2017). Ubiquitin plays a key role in targeting proteins for degradation via polyvesicles (Pelham, 2002a), and selective recognition of autophagic substrates in mammalian cells depends on ubiquitin (Pelham, 2002b). Thus, the shared requirement for targeted substrate degradation by ubiquitin integrates the UPS and ALP into a cohesive degradation system (Zientara-Rytter and Subramani, 2019; Chen R. H. et al., 2019). For example, growth suppressor p27 is regulated by both proteasomal degradation (Borriello et al., 2007; Abbastabar et al., 2018) and the SNX6-mediated endolysosomal pathway (Figure 4F) (Fuster et al., 2010b). Similarly, following TORC1 inhibition, riboproteasomes in yeast are degraded by SNX4-Atg20 and SNX4-ATG41-mediated autophagy (Waite et al., 2016). Although the precise mechanisms remain unclear, these findings suggest that SNX-BAR subfamily members may further modulate UPS activity through their influence on autophagy.

In summary, the SNX-BAR subfamily primarily participates in the ubiquitin-proteasome pathway through modulation by, or interaction with, E3 ubiquitin ligases. Additionally, the SNX-BAR family is a key player in linking the UPS and ALP pathways to form a coordinated degradation system.

## 6 The role of the SNX-BAR subfamily in cytopathological activity and disease

Cargo sorting is essential for maintaining cellular homeostasis, and its dysfunction underlies various diseases, including cardiovascular conditions, neurodegenerative disorders, and cancer (Waite et al., 2016). SNXs, by regulating protein sorting and transport, play crucial roles in membrane transport, organelle movement, cell signaling, and entosis. Dysfunction within the SNX-BAR subfamily can lead to receptor malfunctions and disrupted homeostasis, contributing to disease development (Cullen, 2008d).

SNXs influence cell membrane composition, impacting neuronal excitability, signaling, cognitive responses, and drug resistance (Harashima et al., 2012). Disorders in the SNX-BAR subfamily are linked to several neurodegenerative diseases, including Alzheimer’s disease, Parkinson’s disease, and Down syndrome (Kiani et al., 2003). The role of SNX-BAR in Alzheimer’s disease is detailed in Part II. Notably, SNX5 has been shown to facilitate luteal ptosis in Parkinson’s disease, offering new insights into potential pharmacological targets (Huang et al., 2022). Reduced SNX6 expression impairs synaptic function and spatial memory in CA1 pyramidal neurons (Niu et al., 2017), while SNX8 exacerbates abnormal cholesterol levels and acts as a β-toxic enhancer in Alzheimer’s disease (Muirhead and Dev, 2014). SNX32 is associated with a higher risk of Alzheimer’s disease (Kibinge et al., 2020; Ou et al., 2021), and increased SNX33 expression reduces endocytosis of amyloid precursor protein (Takada-Takatori, 2021).

Recent research highlights the pivotal role of SNXs in cardiovascular disease (Yang et al., 2019), suggesting they could be promising therapeutic targets (Yarmohammadi et al., 2022). SNXs affect blood pressure maintenance through the regulation of G protein-coupled receptors, lipid metabolism, and inflammation (Boulkroun et al., 2008b; Singh et al., 2015). For instance, knockout of SNX1, SNX5, and SNX19 impacts hypertension in animal models (Villar et al., 2013a; Villar et al., 2013b). Lower levels of SNX1 are linked to elevated triglycerides and cholesterol (Burden et al., 2004), while reduced SNX5 expression leads to decreased sodium excretion and increased glucosin and glucose levels, contributing to insulin resistance—a key marker of type 2 diabetes and heart failure (Li et al., 2015; Valera et al., 2003). Such changes heighten cardiovascular disease risk.

Additionally, SNX1 is proposed as a potential tumor suppressor and prognostic marker for gastric cancer (Zhan et al., 2018). SNX2 may serve as a marker for active thyroid cells in both normal and hyperactive thyroid conditions (Kanzawa et al., 2014). Recent findings suggest targeting SNX9 could prevent T cell exhaustion and enhance anti-tumor immunity (Trefny et al., 2023). Overexpression of SNX18 has been linked to increased bacterial internalization and represents a new target for virulence proteins like SopB (Liebl et al., 2017). SNX7 is emerging as a biomarker for diagnosing, prognosticating, and predicting responses to chemotherapy and immunotherapy in liver cancer (Chen et al., 2023). Furthermore, genetic variants reducing SNX7 expression are associated with cognitive dysfunction in psychosis and bipolar disorder (Erhardt et al., 2017).

Given the SNX-BAR subfamily’s critical role in maintaining cellular homeostasis, future research is likely to further elucidate their relationships with neurodegenerative, cardiovascular, and other diseases.

## 7 Conclusion and prospects

This review focuses on the interactions between the SNX-BAR subfamily and the three major pathways involved in maintaining protein homeostasis. This review examines the role of the SNX-BAR subfamily in maintaining protein homeostasis. Like other sorting nexin (SNX) subfamilies, the SNX-BAR subfamily is anticipated to have distinct roles in regulating complex signal transduction and cargo transport, thus enabling precise cellular function regulation. As research on the SNX-BAR subfamily’s involvement in cellular homeostasis advances, our understanding of these proteins continues to expand.

Currently, we have elucidated their fundamental role in protein transport and sorting within the endocytosis pathway, leading to new insights into their contribution to cellular protein quality control. In response to various external stress signals, the SNX-BAR subfamily utilizes conserved evolutionary sequences to direct substrates to specific destinations, either for recycling or degradation. This regulation of intracellular transport by the SNX-BAR subfamily is precisely timed and spatially controlled, highlighting its active role in cellular responses (The summary is shown in Table 1).

**TABLE 1:** SNX-BAR subfamily members involved in three cellular homeostasis pathways. Detailed involvement of 12 SNX-BAR subfamily members in these pathways and their significance in disease and pathophysiology are listed.

SNX-BAR	The endo-lysosomal pathway	The autophngy-lysosome pathway	The ubiquitin-proteasome pathway	Pathophysiological significance
SNX1	Endosomal sorting	Induetion and regulation ofendosomal membrane tubes		High blood pressure; Elevated triglyceride and cholesterol levels; Candidate tumor inhibitors and potential prognostic markers for gastric cancer
SNX2	Fndosomal sorting	Induetion and regulation ofendosomal membrane tubes		Potential markers of active thyroid cells
SNX4	Endosomal recirculation	LC3 is effeetive forlipidation and autophagosome assembly	SNX4 in yeast can further regulate UPSby affecting autophagy	
SNX5	Fndosomal sorting	Mediates autophagyinduced by viral infeetion	Decrease the ubiquitination degradation of HNSCC oncoprotein mediated by FBW7(E3)	Promote luteal ptosis and mediateParkinson’s disease: High blood pressure; Insulin resistance
SNX6	Endosomal sorting		Downregulated Cullin3 (E3) -mediatedubiquitination degradation of PD-L1	Increase the ability of cancer cells to evade attack by the host immune system; Deficits in synaptie function and spatialmemory of CAl pyramidal neurons
SNX7	Endosome sorting and endosone recirculation	Regulating ATG9 tmnsport		New biomarkers for diagnosis, prognosis and prediction of response tochemotherapy and immunotherapy for liver cancer: Genetic varation associated with cognitive dysfunction in psychosis and bipolar disorder
SNX8			Enhance the TRIM28 (E3)-FASNinteraction to promote theubiquitination proteasome degradationofFASN	Exacerbates abnormal cholesterol levelsand mediates the development of Alzheimer’s disease
SNX9	Regulation of endocytosis andendosomal sorting		Interaction with Itch (E3) mediates itsown ubiquitination degradation	Prevent T cell exhaustion and enhance anti-tumor imnunity
SNX18	Regulation of endocytosis andendosounal sorting	Induee autophagosomes toassemble essential proteins	Mibl (E3) regulates SNXl8 recruitment of dynamin 2 and promotesDlll endocytosis	New target of virulence protein (SopB)
SNX30		Regulating ATG9 transport		
SNX32	Endosomal sorting			Increased risk ofAlzheimer’s disease
SNX33	Regulation of endocytosis and endosomal sorting			Reduced endocytosis of amyloid precursor proteins

Members of the SNX-BAR subfamily exhibit diverse cellular localizations and functions, depending on their transport routes. However, research on the mechanisms governing their combined or individual roles in intracellular transport remains limited. Understanding the interactions among SNX family members is an important area for future investigation.

Increasing evidence underscores the significant role of the SNX-BAR subfamily in the progression of various diseases and suggests that specific subfamily members could serve as potential pharmacological targets. Currently, the molecular mechanisms by which the SNX-BAR subfamily influences cardiovascular and neurodegenerative diseases are still in the early stages of exploration and require further development to become effective targets for clinical interventions.
